# Improvements to a GLCM‐based machine‐learning approach for quantifying posterior capsule opacification

**DOI:** 10.1002/acm2.14268

**Published:** 2024-01-23

**Authors:** Chang Liu, Ying Hu, Yan Chen, Jian Fang, Ruhan Liu, Lei Bi, Xunan Tan, Bin Sheng, Qiang Wu

**Affiliations:** ^1^ Department of Ophthalmology Shanghai Sixth People's Hospital Affiliated to Shanghai Jiao Tong University School of Medicine Shanghai China; ^2^ Furong Laboratory Central South University Changsha Hunan China; ^3^ Institute of Translational Medicine Shanghai Jiao Tong University Shanghai China; ^4^ Shanghai University of Sport School of Exercise and Health Shanghai China; ^5^ Department of Computer Science and Engineering Shanghai Jiao Tong University Shanghai China

**Keywords:** cataract surgery, grey level co‐occurrence matrix, machine learning, posterior capsular opacification quantification

## Abstract

**Background:**

Posterior capsular opacification (PCO) is a common complication following cataract surgery that leads to visual disturbances and decreased quality of vision. The aim of our study was to employ a machine‐learning methodology to characterize and validate enhancements applied to the grey‐level co‐occurrence matrix (GLCM) while assessing its validity in comparison to clinical evaluations for evaluating PCO.

**Methods:**

One hundred patients diagnosed with age‐related cataracts who were scheduled for phacoemulsification surgery were included in the study. Following mydriasis, anterior segment photographs were captured using a high‐resolution photographic system. The GLCM was utilized as the feature extractor, and a supported vector machine as the regressor. Three variations, namely, GLCM, GLCM+C (+axial information), and GLCM+V (+regional voting), were analyzed. The reference value for regression was determined by averaging clinical scores obtained through subjective analysis. The relationships between the predicted PCO outcome scores and the ground truth were assessed using Pearson correlation analysis and a Bland–Altman plot, while agreement between them was assessed through the Bland–Altman plot.

**Results:**

Relative to the ground truth, the GLCM, GLCM+C, and GLCM+V methods exhibited correlation coefficients of 0.706, 0.768, and 0.829, respectively. The relationship between the PCO score predicted by the GLCM+V method and the ground truth was statistically significant (*p* < 0.001). Furthermore, the GLCM+V method demonstrated competitive performance comparable to that of two experienced clinicians (*r* = 0.825, 0.843) and superior to that of two junior clinicians (*r* = 0.786, 0.756). Notably, a high level of agreement was observed between predictions and the ground truth, without significant evidence of proportional bias (*p* > 0.05).

**Conclusions:**

Overall, our findings suggest that a machine‐learning approach incorporating the GLCM, specifically the GLCM+V method, holds promise as an objective and reliable tool for assessing PCO progression. Further studies in larger patient cohorts are warranted to validate these findings and explore their potential clinical applications.

## 1 INTRODUCTION

Posterior capsular opacification (PCO) is the most prevalent, long‐term complication following routine cataract surgery and results in decreased visual quality within 2−5 years in 20%−40% of patients, due to lens epithelial cell growth and proliferation.^1^, ^2^ Other common complications include posterior capsule rupture, cystoid macular edema, intraocular lens (IOL) tilt, and decentration.^3^, ^4^, ^5^, ^6^ With advancements in surgical techniques, IOL materials, design, and surface modification, the incidence of PCO has been reduced significantly but not eliminated. Neodymium yttrium aluminum garnet laser (Nd: YAG) capsulotomy is currently the primary treatment option for PCO, which involves the creation of a central opening in the opacified posterior area.^7^ However, this procedure may lead to further complications including ocular inflammation, cystoid macular edema, increased intraocular pressure, retinal detachment, and damage to the IOL.^6^, ^8^ As a result, abundant clinical and experimental studies have been conducted to address the treatment and prevention of PCO, necessitating an objective and standardized measurement for evaluating the efficacy of these interventions.^9^, ^10^, ^11^, ^12^, ^13^, ^14^

In recent years, several methods for quantifying PCO have been reported. Grewal et al developed pentacam tomograms for assessing PCO, but this method requires delineating the region of interest (ROI) for edge detection.^15^ The Scheimpflug video photography system has been utilized to provide reproducible, objective, and quantitative measures of PCO after IOL implantation. Nevertheless, the IOL material significantly affects the density of scattered light, and the optic material affects the quantification of PCO intensity.^16^, ^17^ Optical coherence tomography (OCT) has been also introduced to objectively measure PCO. Nevertheless, its applicability in this domain is restricted since it presupposes a refractive index of 1.36 rather than 1.40, and the measurement encompasses only a coverage area of 1.6 mm^2^.^18^ Additionally, several computerized systems, including Evaluation of Posterior Capsule Opacification (EPCO), Posterior Capsule Opacification (POCO), POCOman, Automated Quantification of After‐Cataract (AQUA), and Aslam Analysis (AA), can partially achieve objective quantification of PCO based on slit‐lamp retroillumination images.^19^, ^20^, ^21^, ^22^, ^23^

In recent years, the integration of artificial intelligence (AI) has emerged as a leading approach in ophthalmology. Our team has been actively involved in the research and development of DeepDR, an AI system for detecting early‐to‐late stages of diabetic retinopathy.^24^ Regarding the quantitative analysis of PCO, only a limited number of software programs (e.g., AQUA II) have introduced machine‐learning based methods to extract texture features and assess the severity of opacification.^25^ Huemer et al utilized code‐free deep learning to improve the diagnostic accuracy of PCO.^26^ However, the effectiveness of these methods can still be improved. The aim of this study was to describe and validate the improvements made to the grey level co‐occurrence matrix (GLCM)‐based machine‐learning approach and compare its validity with clinical assessment for evaluating PCO. The GLCM was employed as a feature extractor, and a supported vector machine (SVM) served as a regressor. Building upon this foundation, we investigated three enhancements at the preprocessing‐level or algorithm‐level.

## 2 MATERIALS AND METHODS

### 2.1 Patients

A total of 100 patients diagnosed with age‐related cataracts and scheduled for phacoemulsification surgery were enrolled in the study. Follow‐up assessments were conducted for a period of 1−3 years after cataract surgery. Anterior segment photographs were captured after mydriasis using a high‐resolution digital coaxial retroillumination photographic system (SRL SL990, C.S.O., Italy). The optical system consists of a slit lamp for observation and imaging, a retrolux illumination module provided by anterior segment flash pack, and beam splitters. The camera system is embedded in the slit lamp system, and the two are integrated. Image capture is performed by pressing a button on the slit lamp handle. The charge‐coupled device (CCD) chip has a geometric resolution of 1624 pixels × 1232 pixels and a radiometric resolution of 24 bits. The exclusion criteria were the presence of corneal abnormalities, history of intraocular surgery, history of ocular or systematic diseases (glaucoma, diabetes, uveitis), and severe intraoperative or postoperative complications (e.g., posterior capsule rupture and IOL tilt). Patients who were unable to achieve pupil dilation to a size of at least 6.5 mm also were excluded from the study. In cases where both eyes of a patient exhibited PCO, only the right eye was included for analysis.

### 2.2 Assessments

Our proposed method for PCO assessment incorporated GLCM as a feature extractor and SVM as a regressor. Building upon this foundation, we introduced three enhancements to further improve the assessment process. The reference value for regression was established based on average clinical scores obtained through subjective analysis.

### 2.3 Pre‐processing

To facilitate accurate analysis, a ROI (i.e., the posterior capsule) was extracted from each RGB image using Adobe Photoshop 2018 (Adobe Inc, Berkeley, CA, USA). Subsequently, the RGB images were transformed into a grey‐level file. Finally, a bounding box was provided to remove irrelevant background.

### 2.4 Subjective analysis

**FIGURE 1:**
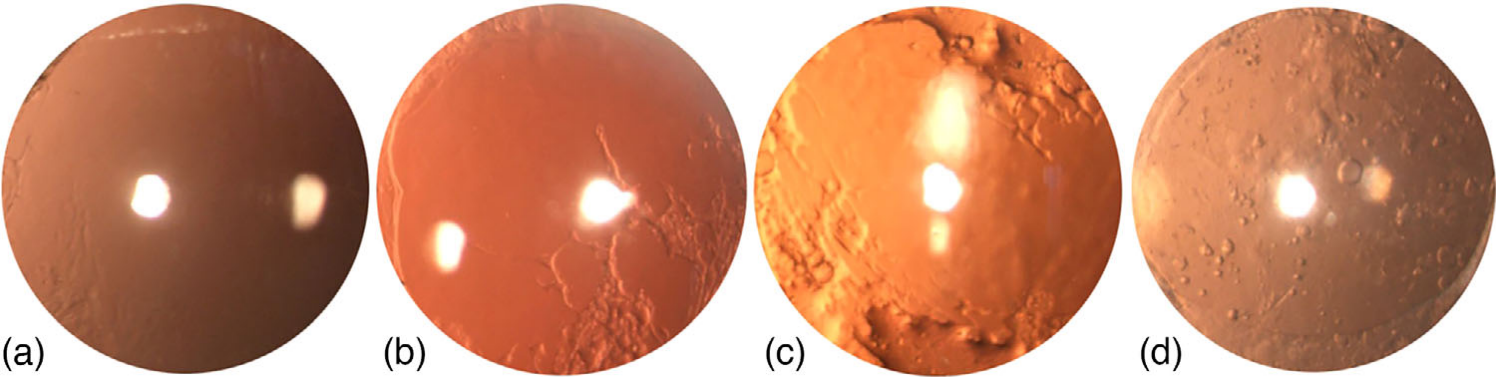
Raw images of eyes with distinctively differing severity of PCO. The intensity of the PCO is minimal (a), mild (b), moderate (c), and severe (d).

Four experienced examiners (two senior clinicians and two junior clinicians) independently evaluated all photographs. Before beginning the grading process, 20 images were viewed by each examiner, and all image scores were decided in one sequence. A week later, a second round of scoring was started to evaluate intraobserver reliability. Figure 1 shows the different intensity of PCO. This method was described by Findl.^22^

### 2.5 Grey level co‐occurrence matrix (GLCM)

The GLCM has been commonly used for texture analysis in medical images.^27^ Given that lesions present in eye images possess specific texture information, the GLCM is employed to extract lesion features. It is defined as the distribution of co‐occurring pixel values at a specified offset within an image. GLCM is the most commonly used method for texture feature extraction. GLCM expresses image texture information by calculating the probability of recurrence of gray‐level structures in the image. Some mathematical models of this co‐production matrix are computed to obtain eigenvalues, which are used to represent some texture features of the image. GLCM can produce comprehensive information regarding the image grayscale in terms of the direction, adjacent interval, magnitude of the change, etc. It is the basis for analyzing the local patterns of an image and its rules of arrangement. For better performance, the input image can be pre‐processed by the Laplacian and Canny enhancement method. From GLCM, five features are generated: angular second moment, contrast, inverse difference moment, entropy, and correlation. By utilizing these features as input, a regressor is trained to accurately predict the ground truth.

### 2.6 Supported vector machine (SVM)

The SVM is a supervised learning model employed for regression analysis. In our study, we utilized a SVM as the regressor to establish a fitting relationship between the GLCM features and the ground truth. The SVM offers advantages over linear regressors by allowing the definition of a tolerable error margin, thereby providing flexibility in the regression process. Notably, the SVM demonstrated strong performance when applied to our dataset.

### 2.7 Improvement 1: ROI extraction

**FIGURE 2:**
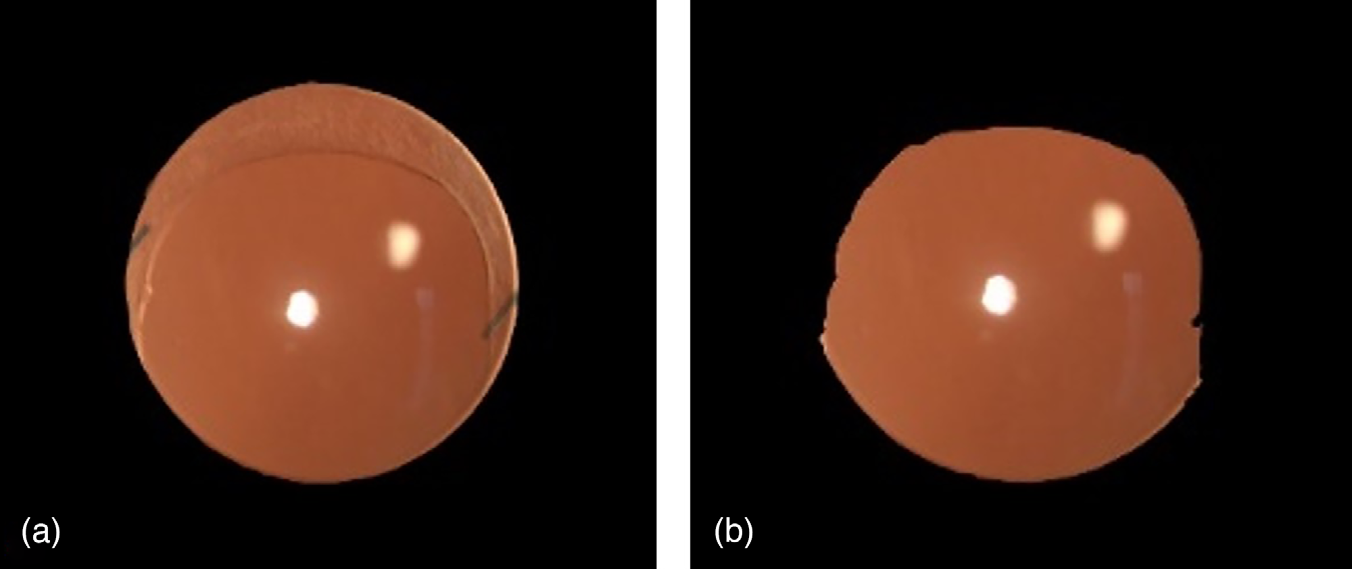
A sample labeled as 1.0 with/without the presence of capsulorhexis was taken: (a) when the capsulorhexis is present, the basic GLCM algorithm predicts a value of 1.95; (b) upon removal of the capsulorhexis, the algorithm predicts a value of 0.99, apparently more correlated to the label.

In a related PCO analysis system, the selection of a ROI within a raw image requires segmentation prior to inputting it into the system. The GLCM approach, which we developed, follows the same process, but we discovered a possible restriction in the accuracy of ROI extraction. Inexperienced clinicians may inadvertently include the IOL and capsulorhexis within the ROI, which can introduce variability and result in higher prediction scores due to the inherent inhomogeneity of these structures. To address this issue, we propose a standardized approach for ROI extraction, defining the ROI as the region encompassing the IOL and capsulorhexis (Figure 2).

### 2.8 Improvement 2: Addition of features of visual axis area

**FIGURE 3:**
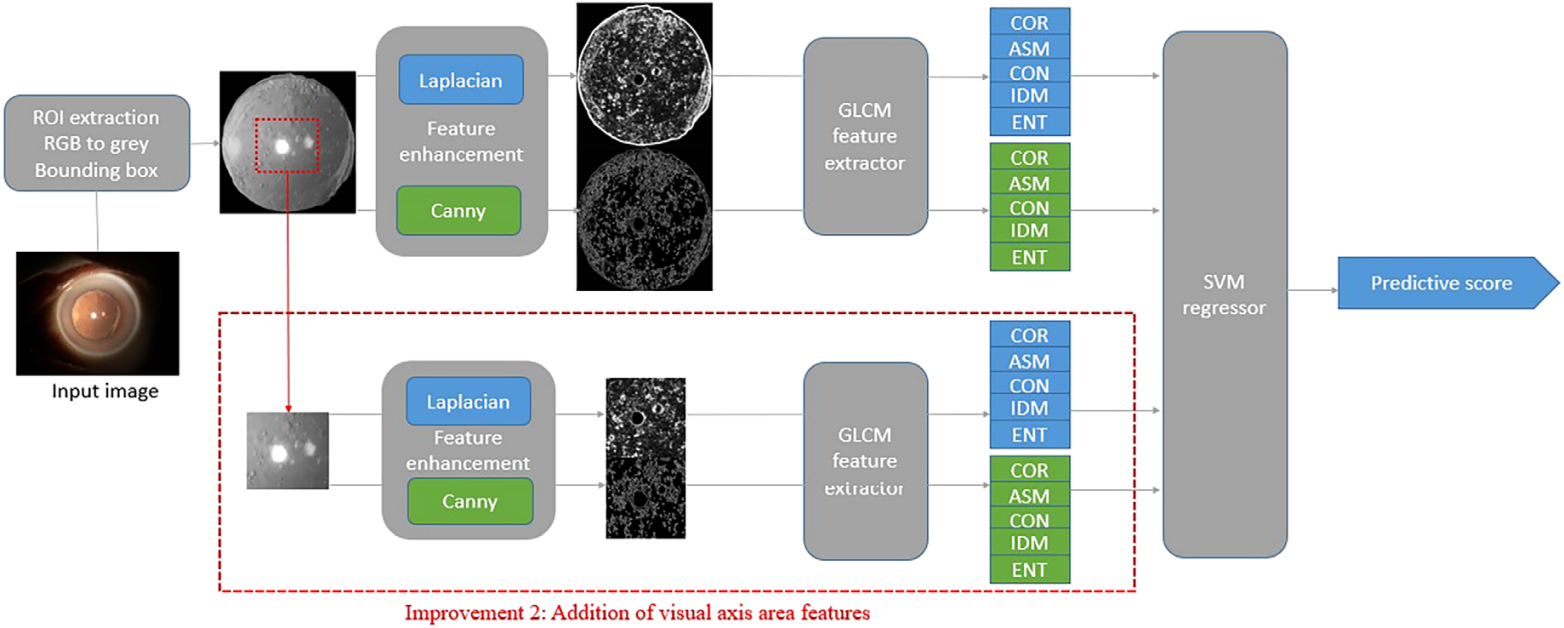
Flow chart of the baseline model and improvement 2.

The assessment of PCO prioritizes the assessment of opacities within the visual axis area. This approach is guided by a diagnostic standard, as opacification in the visual axis area has a greater impact on patients’ visual acuity compared to the peripheral areas. Therefore, we introduced an additional feature in our methodology: GLCM features specifically extracted from the visual axis area (referred to as the GLCM+C method, as depicted in Figure 3). This feature independently captures opacifications within the visual axis, complementing the analysis based on the entire image's GLCM features. As a result, our approach enriches the scale of analysis, encompassing both the global and localized assessment of PCO.

### 2.9 Improvement 3: Regional voting

**FIGURE 4:**
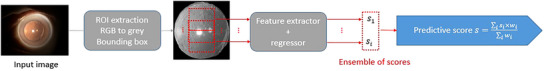
Flowchart of improvement 3. $s_{i}$ indicates the score on the ith region and $w_{i}$ indicates the weight of the ith region.

The third improvement was inspired by two key concepts: (1) ensemble prediction yields superior performance compared to individual predictions; and (2) opacification exhibits a localized distribution rather than a global one. To leverage these ideas, we employed a browsing technique that involves traversing the whole image using a small patch. This allowed us to generate an ensemble of prediction scores using established methods (GLCM feature extractor and SVM regressor). By applying a weighted averaging scheme to this ensemble, we could obtain a final score for the whole image. Furthermore, in alignment with improvement 2, we assigned higher weights to blocks situated within the visual axis regions (as depicted in Figure 4, referred to as the GLCM+V method). In essence, through regional voting, this method integrates predictions from local regions to derive a more dependable and accurate score.

### 2.10 Statistical analysis

Statistical analyses were performed with IBM SPSS Statistics for Windows, version 22.0 (IBM Corp., Armonk, NY, USA). Descriptive statistics were employed to summarize continuous variables as means ± standard deviations. Pearson correlation analysis was used to assess the relationships between the predicted outcome scores and the ground truth. Additionally, Bland–Altman plots were utilized to evaluate the level of agreement between the predicted outcome scores and the ground truth.

## 3 RESULTS

A total of 100 PCO images were included in this study, with 80 photographs randomly assigned to the training and validation datasets, and the remaining 20 images reserved for testing. All the images were graded. During image pre‐processing, the ROI was meticulously isolated, with careful attention to excluding IOL or capsulorhexis. The performances of all three GLCM algorithms were compared, and the average scores generated by each model are presented in Table 1. The averages of the scores provided by the four clinicians were considered as the reference values. Five‐fold cross‐validation was performed for the GLCM and GLCM+C methods, while for GLCM+V, the best model from GLCM was selected and further refined using the regional voting method. The results in Table 1 indicate that the GLCM+V method exhibited the highest correlation and the lowest error on the test set, whereas the GLCM method showed the poorest performance. These findings validate the effectiveness of our improvements to the GLCM method.

**TABLE 1:** Comparison of two improvements made to basic GLCM.

Algorithms	Training set	Validation set	Test set
GLCM	0.797	0.703	0.706
GLCM+C	0.851	0.743	0.768
GLCM+V	–	–	0.829

Abbreviations: C, axial information; GLCM, the basic GLCM algorithm with SVM regressor; V, regional voting method.

The scores provided by the clinicians and those generated by the algorithms were compared using the test set (Table 2). The GLCM+V method demonstrated comparable performance to the experienced clinicians (clinicians 1 and 2) and outperformed the junior clinicians (clinicians 3 and 4).

**TABLE 2:** Comparison of scores obtained by the algorithm and clinicians for the test set.

	Clinician 1	Clinician 2	Clinician 3	Clinician 4	GLCM	GLCM+C	GLCM+V
r	0.825	0.843	0.786	0.756	0.706	0.768	0.829
Mean	0.367	0.35	0.388	0.421	0.448	0.402	0.36
SD	0.222	0.21	0.266	0.265	0.29	0.267	0.219

Abbreviations: C, axial information; GLCM, the basic GLCM algorithm with SVM regressor; V, regional voting method.

**FIGURE 5:**
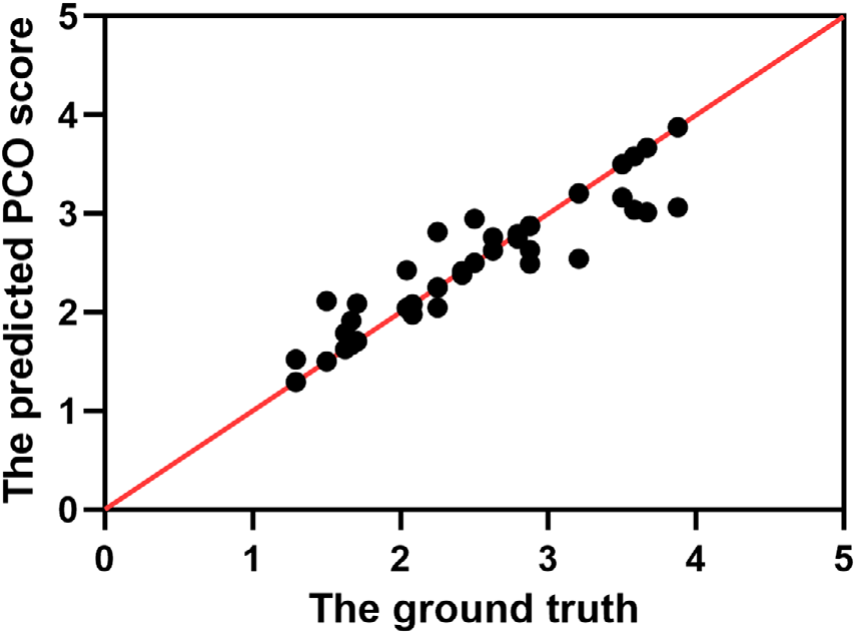
Scatter plot of the predicted PCO scores and the ground truth in the datasets.

**FIGURE 6:**
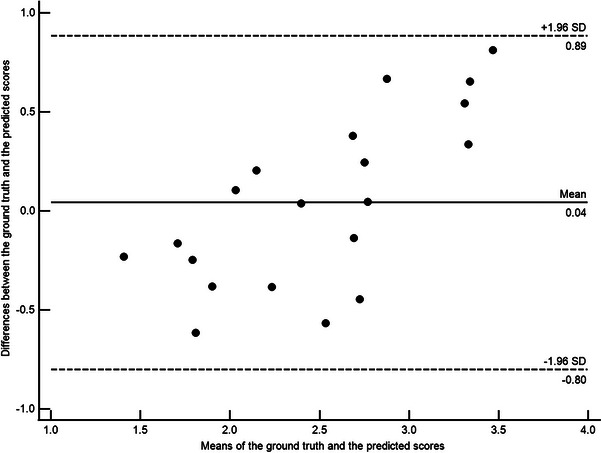
Bland–Altman plot of the predicted PCO scores and the ground truth in the datasets.

The predicted PCO scores obtained using the GLCM+V method were assessed in relation to the ground truth. Pearson correlation analysis revealed a significant relationship between the PCO score predicted by the GLCM+V method and the ground truth (Figure 5, *r* = 0.829, *p* < 0.001). Agreement between predictions and the ground truth was evaluated through Bland–Altman plots, as depicted in Figure 6. The 95% confidence limits of agreement ranged from −0.80 to 0.89, with no significant evidence of proportional bias (*p* > 0.05).

## 4 DISCUSSION

The development of an ideal PCO analysis system requires objectivity, easy of quantification, and sensitivity in calculating PCO scores. Evaluating the surgical techniques, IOL design, research mechanism, and pharmaceutical treatments are crucial in this regard.^17^

To the best of our knowledge, there is no universally accepted gold standard for PCO evaluation. In recent years, several methods have been reported, which can be primarily classified into two types: semi‐automatic and fully automatic objective quantitative analysis systems. Semi‐automatic quantitative analysis systems, such as the EPCO system, Local Co‐occurrence Matrix Mean (LOCOMM) system, and POCOman system, employ coaxial rear reflective illumination photography to acquire posterior capsule opacification (PCO) images. These systems then assess pixel gray values, PCO degree, and area recognition subjectively, enabling an objective and quantitative analysis of PCO.^21^, ^22^, ^28^ Nevertheless, these systems still incorporate certain subjective elements, potential observer bias, and dependency on hardware support. Automated quantitative analysis systems, such as Scheimpflug slit‐lamp photography, POCO software, AQUA system, and AA system, utilize structural analysis techniques with rear reflective illumination and coaxial light to process high‐definition PCO images. The objective is to eliminate reflective points and improve image contrast.^20^, ^22^, ^29^, ^30^ The texture segmentation method used by POCO and AA can address challenges in identifying areas with uniform cloudiness. As well as subtle changes in pixel density, especially for large areas with thin and dense fiber clouding. Nevertheless, as per the existing literature, certain software systems mentioned earlier exhibit early or moderate PCO evaluation saturation. They demonstrate a weak correlation with clinical slit lamp evaluation and possess limited capacity to differentiate between various types and morphologies of PCO turbidity.

Texture analysis has been widely used in computer vision for medical image assessment and classification and offers a valuable approach for analyzing textured and non‐textured areas.^17^ Among texture analysis methods, the GLCM is commonly employed, providing quantitative descriptions of texture through parameters like entropy and contrast. Nevertheless, the GLCM can be affected by image brightness, requiring high‐quality raw images for accurate analysis.^27^ Moreover, the GLCM tends to extract features uniformly across the entire image, potentially compromising its performance by confusing artifacts with lesions. Extracted GLCM‐features on whole images can be rough, implying feature extraction on a local scale.^25^ Hence, we described and validated a new automated method for PCO assessment in retroillumination images based on GLCM.

In the present study, the modified GLCM system was used for qualitatively and quantitatively evaluating PCO. Our results demonstrated a significant correlation between clinical grading estimation and GLCM+V (*r* = 0.829). Barman et al. developed an objective assessment method, utilizing texture segmentation and co‐occurrence matrix, which also showed a significant correlation with clinical values.^20^ Findl et al. compared four different PCO evaluation methods and found that the subjective grading system exhibited the strongest correlation with automated quantification for PCO.^22^ Findl et al. also reported a significant correlation between subjective and objective grading methods, which was also observed in the GLCM method.^25^

With the recent development of deep learning and convolutional neural network (CNN) techniques, investigators have also attempted to use CNNs for feature extraction from various medical images.^31^, ^32^, ^33^ Benyahia et al explored the use of 17 commonly used pre‐trained CNN architectures as feature extractors for feature extraction and then used 24 classifiers for skin lesions classification task.^34^ Want et al proposed to reconstruct CT images with CNN networks by fusing information derived from a few projections, such that minimized the exposure to radiations while increasing the scanning speed.^35^ However, the performance of deep learning‐based methods is heavily reliant on the availability of annotated medical data, which is usually difficult to acquire due to various ethics constraints and expensive data acquisition procedures. In addition, deep learning‐based methods usually work as a black box, such that will have difficulties in interpreting the extracted features.

The present study has some limitations. Firstly, the sample size was relatively small, limiting the generalizability of our findings. We plan to evaluate the proposed method in a larger clinical dataset in our future study. Secondly, we only compared clinical subjective grading, and the results from other software programs and algorithms were not considered in our experiments. In our future studies, we plan to use deep learning‐based methods to enhance the role of AI in the quantitative evaluation of PCO.

## 5 CONCLUSION

Our enhanced approach, utilizing the GLCM machine‐learning approach, provides results that strongly correlate with clinical assessment. The algorithm we presented provides an objective scoring system. We have demonstrated the system's effectiveness for both novice clinicians and seasoned practitioners, where the system can serve as a viable alternative tool with similar levels of accuracy. Additionally, our method demonstrates reliability in quantifying PCO, making it a valuable asset for experimental investigations and clinical research.

## AUTHOR CONTRIBUTIONS

Qiang Wu and Bin Sheng contributed to the conception of the study; Chang Liu performed the experiment and wrote the manuscript; Ying Hu contributed significantly to analysis and manuscript preparation; Jian Fang and Yan Chen performed the data analyses; Ruhan Liu helped perform the analysis with constructive discussions; Lei Bi and Xunan Tan helped revise the manuscript. All authors read and approved the final version of the manuscript.

## CONFLICT OF INTEREST STATEMENT

The authors declare that they have no conflict of interests.

## ETHICAL STATEMENT

The study was conducted following the principles of the Declaration of Helsinki. All patients signed an informed consent form, and all private information was removed. The study was approved by the Institutional Ethical Committee of the Shanghai Jiao Tong University‐Affiliated Sixth People's Hospital (2019‐KY‐045(K)).

## Data Availability

The datasets generated and analyzed in the present study are available from the corresponding author upon reasonable request.
